# Supplementary material for: Optimization of the dosage of chopped basalt fibers in asphalt pavement surface course materials for semi-rigid base with functional requirements
Source: PLoS One. 2024 Jul 29;19(7):e0307438. doi: 10.1371/journal.pone.0307438 (PMC11285914; doi:10.1371/journal.pone.0307438)
Supplement: Attachment — Submitted filename: Dr. Rana Amir.pdf [file pone.0307438.s003.pdf]

After a full review of the research ((Optimization of the Dosage of Chopped Basalt Fibers in Asphalt Pavement Surface Course Materials for Semi-rigid Base with Functional Requirements)) , it was concluded that:

1. The research idea was previously proposed, although it is considered good.

2. It is preferable to add more tests for evaluation.

3. The research is considered acceptable in terms of the number of sources and their modernity.

4. The language of the research is scientifically good.

So we recommend accepting the research.

My respects and appreciation.
