# Supplementary material for: Optimization of the dosage of chopped basalt fibers in asphalt pavement surface course materials for semi-rigid base with functional requirements
Source: PLoS One. 2024 Jul 29;19(7):e0307438. doi: 10.1371/journal.pone.0307438 (PMC11285914; doi:10.1371/journal.pone.0307438)
Supplement: Attachment — Submitted filename: Optimization of the use of basalt fiber in asphalt pavement.docx [file pone.0307438.s001.docx]

**RESEARCH ARTICLE**

Optimization of the Dosage of Chopped Basalt Fibers in Asphalt Pavement Surface Course Materials for Semi-rigid Base with Functional Requirements

Xiangbing Xie1,*,Yahui He1 , Chenchen Liu1, Kaiwei Wang 1, Zhezhe Fan2*, Huixia Li3,Jinggan Shao4

^1^	School of Civil Engineering and Architecture, Zhengzhou University of Aeronautics, Zhengzhou 450046, Henan, China

^2^ School of Civil and Transportation Engineering,Henan University of Urban Construction,Pingdingshan 467036, Henan, China

^3^ School of Civil Engineering, Fujian University of Technology, Fuzhou, Fujian, 350108, China,

^4^ Henan College of Transportation Engineering Technology Group Co., Ltd. Green High-performance Material Application Technology Transportation Industry R&D Center, Zhengzhou 450018, Henan, China;

^*^ Correspondence: xiexiangbing.good@163.com (X. Xie); fanzhezhe0610@163.com (Z. Fan)

**Abstract:** How to select suitable pavement materials for asphalt pavements according to the functional requirements of layers is still the focus of research by scholars in various countries. Basalt fibers are excellent in improving high-temperature stability and are mainly used to improve the resistance to low-temperature cracking and water damage in upper-layer asphalt mixtures. However, in the middle and lower layers of mixtures, their effects on resistance to high-temperature rutting and fatigue cracking are limited. Therefore, the optimum basalt fiber blending in different layers to enhance road performance was investigated and optimized with design specifications. In this study, nine asphalt pavement structures with different dosages of basalt fibers were combined by an orthogonal test method. The optimal structure was determined by considering factors such as fatigue life and overloading using the Abaqus finite element model. The results of the study showed that the fiber dosage had a small effect on the bending subsidence of the road surface and the location of the tensile strain in the lower layer, and the shear stresses mainly occurred at the outer edges of the loads. The optimum dosage of basalt fiber asphalt pavement structures based on fatigue life and overload was 0.3% for the upper layer, 0.1% for the middle layer, and 0.3% for the lower layer. The optimum structure based on a strong base thin-surfaced semi-rigid base layer was 0.3% for the upper layer and 0.1% for the middle layer. This provides a useful reference for the design of basalt fiber asphalt pavement.

**Keywords:** asphalt pavement surface course; basalt fiber; functional requirements ;optimization study

1. Introduction

Asphalt concrete pavement, known for its comfortable driving, low noise, and easy maintenance, has been widely applied in highway construction. While asphalt mixtures serve as the primary structural layer to bear pressure, it is essential to study their roles in different layers to comprehensively understand their performance. Additionally, in practical applications, various environmental factors and vehicle loads lead to asphalt pavement distress, such as ruts, potholes, and cracks[1-2], significantly impacting the lifespan and performance of the asphalt pavement[3].

To cope with the poor performance of mixtures, road builders actively use various additives for improvement[4-6]. Among the many additives, fibers, including lignin fibers, basalt fibers, steel fibers[7], carbon fibers[8], glass fibers[9], and polyester fibers[10], have been recognized for effectively enhancing the performance of asphalt mixtures. However, basalt fiber, as an environmentally friendly fiber material[11], is used as a reinforcing material in a variety of fields because of its cost-effective and environmentally friendly advantages, as well as its resistance to acids and alkali, high strength, and resilience to high and low temperatures[12-13].

Today, with the increase in traffic volume, basalt fiber asphalt mixture is considered an effective material for enhancing road performance, including fatigue resistance, resistance to water damage, and crack resistance[14]. For example, the optimum mixing amount of basalt fiber in stone mastic asphalt(SMA) concrete is 0.35%, whose dynamic stability and Marshall stability performance are better compared to other fibers, especially in the high-temperature stability of the most outstanding performance [15-16]. Using the asphalt concrete(AC-13C) mixture, various amounts of basalt fibers (0.1%, 0.2%, 0.25%, 0.3%, 0.35%) were added, and high-temperature rutting tests were conducted. The results indicate that the optimal fiber content is 0.25% for achieving the best road performance[17]. For the AC-13C asphalt mixture, the optimal basalt fiber content is 0.3%, resulting in an approximately 35% improvement in dynamic stability at the optimal dosage[18-19]. The overall performance of fiber asphalt mixtures was better when basalt fibers, lignin fibers, and polyester fibers were added to AC-20 asphalt mixtures at 0.3%, 0.4%, and 0.2%, respectively[20]. Basalt fiber effectively improves the low-temperature performance of asphalt mixtures, making them more adaptable to low-temperature environments. According to the low-temperature performance, the optimal basalt fiber content of AC-20 mixtures is 0.3%, at which the network structure of basalt fibers enhances the integrity of asphalt mixtures and effectively retards the expansion of microcracks [21-22]. Overall, adding 0.3% basalt fibers significantly enhances the dynamic stability, maximum failure strain, high-temperature stability, water stability, and low-temperature crack resistance of the asphalt mixture, optimizing the overall road performance[23-28].

In the design optimization of pavement structure, Utilizing the BISAR3.0 pavement structure design and analysis software, as well as the Hills method, an analysis of semi-rigid base asphalt pavement was conducted by varying factors such as surface layer thickness, modulus, traffic load, and environmental temperature. The primary structural function of the upper layer is to resist low-temperature cracking, while the middle layer focuses on high-temperature resistance against rutting, and the lower layer primarily addresses fatigue cracking resistance[29-32]. Employing fiber optic grating testing technology, a three-dimensional dynamic strain response analysis of asphalt pavement under typical truck loads was performed, revealing significant compressive stress impact on the upper and middle layers, while the lower layer exhibited a cyclic "compression-tension" stress variation, making it more susceptible to fatigue cracking[33]. Structural analysis using ANSYS finite element software showed that the middle layer of the asphalt pavement primarily bears the shear stress in the rut resistance zone, whereas the lower layer primarily withstands the tensile stress due to the load on the asphalt pavement, emphasizing its role in fatigue crack resistance[34]. Consequently, the functional requirements of asphalt pavement in China involve resistance to surface cracking, skid resistance, thermal shrinkage stress, and water damage in the surface layer. The middle layer is designed for high-temperature stability, while the lower layer is engineered for fatigue crack resistance and high-temperature stability.

In summary, through the research on the optimization of the performance of basalt fibers in asphalt mixtures, as well as the stress analysis of the pavement structure, this paper provides an in-depth insight into the design and study of basalt fiber asphalt pavement structure, which provides a reliable theoretical basis for improving the performance of the pavement, prolonging the service life, and reducing the maintenance cost.

2. Materials and Methods

**2.1. Selection of raw materials**

70# modified asphalt was used in the experiment, and its performance indicators were as follows:

**Table 1.** Performance indicators of modified asphalt.

| **Parameters** | **Requirements** | **Experimental results** | **Experimental method** |
| --- | --- | --- | --- |
| Penetration (25%)/0.1 mm | 40-60 | 53.4 | GB/T 4509 |
| Softening point/ °C | ≥ 60 | 79 | GB/T 4507 |
| Ductility (5 ℃,5 cm/min)/cm | ≥ 20 | 27.4 | GB/T 4508 |
| Penetration index PI | -1.5-1 | 0.16 |  |
| Solubility/% | ≥ 99.0 | 99.5 | GB/T 11148 |
| Flashpoint/ °C | ≥ 230 | 281 | GB/T267 |

Chopped 6mm/17 μm basalt fiber was used in this experiment, and its various performance indicators are shown in Table 2.

**Table 2.** Performance indexes of chopped basalt fiber.

| **Parameters** | **Requirements** | **Experimental results** |
| --- | --- | --- |
| Breaking strength/MPa | ≥ 1200 | 1260 |
| Elongation at break/% | ≤ 3.1 | 2.65 |
| Oil absorption/% | ≥ 50 | 53 |
| Flammability content% | 0.1-1.0 | 0.5 |
| Moisture content% | ≤ 0.2 | 0.14 |
| Flammability | open flame otherwise | open flame otherwise |

***2.2. Selection of influencing factors***

Basalt fiber reinforced the gap-graded asphalt concrete SMA-16(BFSMA-16), basalt fiber reinforced the dense gradation asphalt concrete AC-20(BFAC-20), and basalt fiber reinforced asphalt-treated permeable base ATB-25(BFATB-25) were selected as factors according to research results from the literature[15，20，21，24，25], the dosage of BFSMA-16 was 0.1%, 0.2%, or 0.3%, the dosage of BFAC-20 was 0.1%, 0.2% %, or 0.3%, and the BFATB-25 content was 0.1%, 0.2%, or 0.3%. The level number was selected according to the principle that the number of factors ≤ the number listed in the orthogonal table, and the level number of the factor was consistent with the level number corresponding to the orthogonal table, which was selected as (). Research on optimal pavement structure was conducted, see Table 3 for the test scheme.

**Table 3.** Orthogonal experimental design scheme.

| **Test** | **Influencing factors** | | | |
| --- | --- | --- | --- | --- |
|  | **BFSMA-16(A) /%** | **BFAC-20(B) /%** | **BFATB-25(C)/ %** | **Blank (D)** |
| 1 | 0.1 | 0.1 | 0.1 | 1 |
| 2 | 0.1 | 0.2 | 0.2 | 2 |
| 3 | 0.1 | 0.3 | 0.3 | 3 |
| 4 | 0.2 | 0.1 | 0.2 | 3 |
| 5 | 0.2 | 0.2 | 0.3 | 1 |
| 6 | 0.2 | 0.3 | 0.1 | 2 |
| 7 | 0.3 | 0.1 | 0.3 | 2 |
| 8 | 0.3 | 0.2 | 0.1 | 3 |
| 9 | 0.3 | 0.3 | 0.2 | 1 |

**2.2. Test method**

**2.3.1. Dynamic modulus test**

The stress process experienced by the asphalt pavement surface layer under traffic loads is highlighted, emphasizing the use of a dynamic loading model for a more accurate analysis of the asphalt pavement's viscoelastic properties and stress conditions. In the NCHRP program, the Simple Performance Tester (SPT) is employed to determine the dynamic modulus of asphalt mixtures under uniaxial compression, allowing for the assessment of material characteristics and providing a basis for the design of asphalt pavement materials and structures.

According to Chinese national standards, the mineral gradation and optimal asphalt-to-aggregate ratio of SMA-16, AC-20, and ATB-25 mixtures are determined. The content of basalt fiber is then varied at four levels: 0%, 0.1%, 0.2%, and 0.3%. To measure the dynamic modulus, cylindrical specimens are prepared using a gyratory compactor, with metal pins attached to the specimens. The specimens are then placed in a constant temperature chamber for insulation. Finally, the measurement is conducted through the testing procedure, and the corresponding process is illustrated, as shown in Figure 1.

|   a. Paste metal nails |   b. Test piece insulation |
| --- | --- |
|   c. Determination of dynamic compression modulus |   d, Data logging |
| **Figure 1.** Dynamic modulus test process diagram | |

2.3.2. Establishment of the 3D finite element model

The selected semi-rigid base asphalt pavement structure form is shown in Figure 2, which is the traditional pavement structure for highways. The viscoelastic characteristics of the upper layer SMA-16, middle layer AC-20, and lower layer ATB-25 are represented using the Viscoelastic model in ABAQUS.

Asphalt mixtures are viscoelastic materials whose mechanical behavior is significantly dependent on time. The Burgers four-element mechanical model reveals the viscoelastic properties of mixtures. When deformation is applied for a very short period, the mix shows significant viscosity, however, the viscosity diminishes when the same deformation is applied for a long period. The creep and stress relaxation properties exhibited by the mixes over the experimental time range caused the mechanical modulus of the mixes to decrease with time. The generalized Pliny series model, which is commonly used in the viscoelastic constitutive relationship of asphalt mixtures, succinctly reflects the creep and stress relaxation properties of the mixes.

In ABAQUS finite element software, the viscoelastic model uses the shear modulus of the Proni series to represent the change in material with time. The input parameters include a set of Prony series coefficients and their corresponding relaxation times. The shear modulus can be calculated from the relaxed modulus using Equation (1), which is expressed as follows:

$$G\left( t \right)=\frac{E\left( t \right)}{2\left( 1+\mu\right)} (1)$$

Formula:$G\left( t \right)$－shear modulus; $E\left( t \right)$－relaxed modulus;$\mu$－Poisson's ratio.

The shear modulus ratio obtained from the instantaneous shear modulus GT and normalized shear modulus GT as experimental data for fitting the Prony series in ABAQUS is represented as g(t).

$$g\left( t \right)=1-\sum_{i=1}^{N} g_{i}\left( 1-e^{-t/\tau_{i}} \right) (2)$$

Formula: N－The number of terms in the Prony series;－Prony level parameters;－latency.

The broad sense Prony series and the generalized Kelvin model share the same analytical form, with the distinction that the former is not constrained by the number and interval of terms, allowing for the experimental results to be freely specified. A set of delay times, encompassing the entire experimental process, is chosen. The parameters of the Prony series in Equation (2) are obtained using the nonlinear least squares curve fitting method.

Generally, when the Prony level is taken as 5 terms, the measured shear modulus ratio can be fitted to within 0.01 of the mean squared error, to more accurately simulate the actual use of 5 Prony level for three kinds of asphalt mixtures parameter fitting. The fitting results of the Prony levels for the three mixtures are shown in Table 4, 5 and 6. WLF parameters are shown in Table 7.The compressive modulus of the base layer was 7500 MPa, and the compressive modulus of the subgrade was 7500 MPa.

**Figure 2.** Traditional pavement structure.

Table 4. Prony Series Parameters for SMA-16

| Parameters | Values | Parameters | Values |
| --- | --- | --- | --- |
| *g_1_* | 0.4368 | *τ*_1_ | 1.26E-05 |
| *g_2_* | 0.2742 | *τ*_2_ | 3.96E-04 |
| *g_3_* | 0.1921 | *τ*_3_ | 6.12E-03 |
| *g_4_* | 0.1023 | *τ*_4_ | 0.1341 |
| *g_5_* | 0.0562 | *τ*_5_ | 12.875 |

Table 5. Prony Series Parameters for AC-20

| Parameters | Values | Parameters | Values |
| --- | --- | --- | --- |
| *g_1_* | 0.3342 | *τ*_1_ | 1.05E-05 |
| *g_2_* | 0.2931 | *τ*_2_ | 1.51E-04 |
| *g_3_* | 0.2723 | *τ*_3_ | 3.50E-03 |
| *g_4_* | 0.0814 | *τ*_4_ | 0.1216 |
| *g_5_* | 0.0345 | *τ*_5_ | 6.8132 |

Table 6. Prony Series Parameters for ATB-25

| Parameters | Values | Parameters | Values |
| --- | --- | --- | --- |
| *g_1_* | 0.3911 | *τ*_1_ | 2.71E-05 |
| *g_2_* | 0.2503 | *τ*_2_ | 6.69E-04 |
| *g_3_* | 0.2421 | *τ*_3_ | 9.54E-03 |
| *g_4_* | 0.1132 | *τ*_4_ | 0.2013 |
| *g_5_* | 0.0325 | *τ*_5_ | 10.893 |

Table 7. WLF Equation Coefficients

| Parameters | $C_{1}$ | $C_{2}$ | R |
| --- | --- | --- | --- |
| SMA-16Values | 9.735 | 122.624 | 0.99912 |
| AC-20Values | 12.646 | 133.965 | 0.99821 |
| ATB-25Values | 18.423 | 162.879 | 0.99142 |

The moving load adopted the standard axle load used in the current structural design of asphalt pavement in China, and the load adopted the double-circle vertical uniform load, that is, the standard axle load of 100 kN for a single axle load of the double-wheel set, the ground pressure of the tire was 0.7 MPa, the radius of action of the load was r=0.1065 m, and the distance between the centers of the double circles was 3r=0.3195 m. The X-direction was the driving direction, the Y-direction was the road cross-sectional direction, and the Z-direction was the road depth direction; the calculation points were selected according to the corresponding provisions in JTG D50-2017 "Code for Design of Highway Asphalt Pavement", as shown in Figure 3 below.

**Figure 3.** Calculation point.

The three-dimensional finite element model was a five-layer structure of asphalt pavement, and the total thickness of the pavement was 76 cm. In the upper surface of the pavement role of the traffic load, the model length, width, and height were 6 m, 6 m, and 3 m, respectively. The mesh was divided into 0.1 × 0.1, and the load area coded division was 0. 05 × 0.05, and the calculation unit for the 8-node hexahedral reduction integral unit (C3D8R), the model and mesh division are shown in Figure 6. The model and mesh division are shown in Figure 4, where the X-direction is the direction of travel of the pavement structure, and the Y-direction is the depth direction of the pavement structure.

To simplify the calculation, a standard axle load of 0.7 MPa was chosen. The specific load locations and boundary conditions are shown in Figure 5.

**Figure 4.** Pavement structure model and grid division.

**Figure 5.** Load action position and boundary conditions.

3. Test results and analysis

**3.1. Dynamic modulus**

Taking the SMA-16 test as an example, the relevant experimental test conditions and results are as follows:

(1) Temperature: The temperature range controlled by the environmental chamber that comes with the SPT tester is from 5 °C to 50 °C. Considering the research objectives of this study, the test temperatures of 5 °C, 20 °C, 30 °C, 40 °C and 50 °C were selected.

(2) Frequency: Considering the actual working frequency of the asphalt mixtures and the duration of the test, the loading frequencies of 0.1 Hz, 0.5 Hz, 1 Hz, 5 Hz, 10 Hz, and 25 Hz were selected.

(3) Waveform: It is believed that the Haversine waveform corresponds to the actual vehicle load waveform experienced by the pavement. Additionally, it can be easily achieved using existing testing equipment. Therefore, the following Haversine waveform was adopted in this study：

$$F=\frac{q}{2}(1-\cos(2\pi ft ))$$

In the equation, "f" represents the excitation frequency of the vehicle load, which is related to the driving speed, natural frequency of the vehicle, etc. It is a key parameter for conducting pavement mechanical response analysis.

(4) Number of Loading Cycles: The number of repeated loading cycles under different load frequencies is shown in Table 8.

Table 8: Number of repetitions of loading cycles at different load frequencies

| Frequency（Hz） | 25 | 10 | 5 | 1 | 0.5 | 0.1 |
| --- | --- | --- | --- | --- | --- | --- |
| Number of Repetitions（count） | 200 | 200 | 100 | 20 | 15 | 15 |

(5) The test results are shown in Figure 2, 3.

(a)Different Temperatures	(b)Different Frequency

Figure 6. Dynamic Modulus Curves of SMA-16 at Different Frequencies and Temperatures

According to Figure 6(a), it can be observed that with the increase in frequency, the dynamic modulus continuously increases, and the increase is more pronounced at lower temperatures, with the smallest range of dynamic modulus variation occurring at 60°C. As shown in Figure 6(b), with the rise in temperature, the dynamic modulus gradually decreases, and the reduction becomes more significant at higher frequencies. In summary, when frequency and temperature are coupled, the influence of temperature on dynamic modulus is far greater than the influence of frequency.

(a)Different Temperatures	(b)Different Frequency

Figure 7. Phase Angle Curves of SMA-16 at Different Frequencies and Temperatures

As indicated in Figure 7, at low temperatures (5°C to 20°C), the phase angle increases with rising temperature and decreases with increasing frequency. The lower the temperature, the faster the decrease in phase angle with increasing frequency. At relatively higher temperatures (40°C to 50°C), the phase angle decreases with increasing temperature and increases with frequency.

**3.2 Structural analysis of basalt fiber refreinforced asphalt pavement surface**

Since the upper layer of the pavement was mainly resistant to low-temperature cracking, the middle layer was mainly stable at high-temperature, and the lower layer was resistant to fatigue cracking, we used the road surface deflection, maximum shear stress, and tensile strain at the bottom of the asphalt layer as evaluation indicators to select the optimum combination of pavement structure layers.

From the dynamic modulus, the pavement surface deflection, shear stress and asphalt base tensile strain were obtained at different positions. The results of the calculations are shown in Figure 8, Figure 9and Figure 10 below.

**Figure 8.** The deflection of the upper layer of the road surface under different dosages.

**Figure 9.** The maximum shear stress of the middle surface layer under different dosages (kPa).

**Figure 10.** The maximum tensile strain at the bottom of the layer under different dosages (×10^(-6)).

Figure 8 shows that the maximum value of the deflection of the road surface at different positions for asphalt mixtures with different dosages was at point B at the single-circle load center. In Figure 9, for asphalt mixtures with different dosages, most of the maximum shear stress at different locations was at point B at the single-circle load center. Only when the fiber content of the lower layer was 0.3% was the maximum shear stress at Point A at the outer edge of the circular load. As shown in Figure 10 above, for the tensile strain at the bottom of the layer under different dosages of asphalt mixtures at different positions, the maximum value was at point B at the center of the single circular load.

**3.3 Orthogonal test result analysis**

**3.3.1 Range analysis**

From Figure 8, Figure 9, and Figure 10 above, the calculation results for the maximum road surface deflection, maximum shear stress and maximum tensile strain at point B were filled in Table 9, and for each index, the data of each factor level , , and the corresponding average value , , and the range of each column were calculated and filled in Table 9.

**Table 9.** Orthogonal test range analysis table.

| **Test No.** | | **Influencing factors** | | | | **Test index** | | |
| --- | --- | --- | --- | --- | --- | --- | --- | --- |
|  |  | **BFSMA-16(A) /%** | **BFAC-20(B) /%** | **BFATB-25(C)/ %** | **Blank (D)** | **Road surface deflection**  **(mm)** | **Maximum shear stress (kPa)** | **The maximum tensile strain at the bottom of the layer (×10^(-6))** |
| 1 | | 0.1 | 0.1 | 0.1 | 1 | 13.44 | 168.7 | 6.984 |
| 2 | | 0.1 | 0.2 | 0.2 | 2 | 13.30 | 167.6 | 7.278 |
| 3 | | 0.1 | 0.3 | 0.3 | 3 | 13.15 | 165.3 | 7.490 |
| 4 | | 0.2 | 0.1 | 0.2 | 3 | 13.33 | 164.9 | 7.247 |
| 5 | | 0.2 | 0.2 | 0.3 | 1 | 13.16 | 163.6 | 7.413 |
| 6 | | 0.2 | 0.3 | 0.1 | 2 | 13.18 | 175.5 | 7.310 |
| 7 | | 0.3 | 0.1 | 0.3 | 2 | 13.23 | 161.6 | 7.300 |
| 8 | | 0.3 | 0.2 | 0.1 | 3 | 13.17 | 173.1 | 7.345 |
| 9 | | 0.3 | 0.3 | 0.2 | 1 | 13.15 | 171.0 | 7.511 |
| Road surface deflection | $K_{1j}$ | 39.89 | 40 | 39.79 | 39.75 |  | | |
|  | $K_{2j}$ | 39.67 | 39.63 | 39.78 | 39.71 |  |  |  |
|  | $K_{3j}$ | 39.55 | 39.48 | 39.54 | 39.65 |  |  |  |
|  | $\bar{K}_{1j}$ | 13.297 | 13.333 | 13.263 | 13.25 |  |  |  |
|  | $\bar{K}_{2j}$ | 13.223 | 13.21 | 13.26 | 13.237 |  |  |  |
|  | $\bar{K}_{3j}$ | 13.183 | 13.16 | 13.180 | 13.217 |  |  |  |
|  | $R_{j}$ | 0.114 | 0.173 | 0.083 | 0.033 |  |  |  |
| Maximum shear stress | $K_{1j}$ | 501.6 | 495.2 | 517.3 | 503.3 |  |  |  |
|  | $K_{2j}$ | 504 | 504.3 | 503.5 | 504.7 |  |  |  |
|  | $K_{3j}$ | 505.7 | 511.8 | 490.5 | 503.3 |  |  |  |
|  | $\bar{K}_{1j}$ | 167.200 | 165.067 | 172.433 | 167.867 |  |  |  |
|  | $\bar{K}_{2j}$ | 168.000 | 168.100 | 167.833 | 168.233 |  |  |  |
|  | $\bar{K}_{3j}$ | 168.567 | 170.600 | 163.500 | 167.767 |  |  |  |
|  | $R_{j}$ | 1.367 | 5.533 | 8.933 | 0.466 |  |  |  |
| Maximum tensile strain | $K_{1j}$ | 21.752 | 21.531 | 21.639 | 21.908 |  |  |  |
|  | $K_{2j}$ | 21.97 | 22.036 | 22.036 | 21.888 |  |  |  |
|  | $K_{3j}$ | 22.156 | 22.311 | 22.203 | 22.082 |  |  |  |
|  | $\bar{K}_{1j}$ | 7.251 | 7.177 | 7.213 | 7.303 |  |  |  |
|  | $\bar{K}_{2j}$ | 7.323 | 7.345 | 7.345 | 7.296 |  |  |  |
|  | $\bar{K}_{3j}$ | 7.385 | 7.437 | 7.401 | 7.361 |  |  |  |
|  | $R_{j}$ | 0.134 | 0.260 | 0.188 | 0.065 |  |  |  |

The range $R_{j}$ was not equal, indicating that the changes in the levels of each factor had different effects on the experimental results, and the larger the range $R_{j}$ was, the greater the impact of the change in the factor level on the experimental results. In Tables 9, in the structure of the pavement made of asphalt modified with basalt fiber, under the continuous state between layers, the influence of the basalt fiber content of the middle surface layer on the deflection of the road surface was greater than that of the upper layer and greater than that of the lower layer. For the lower layer of basalt, the effect of the fiber content on the maximum shear stress was greater than that of the middle surface layer and greater than that of the upper layer. The effect of the basalt fiber content of the middle surface layer on the maximum tensile strain at the bottom of the layer was greater than that of the bottom layer and greater than that of the upper layer. If it was smaller than the range of all factors, it meant that there was no interaction between the factors, and yet it meant that the abovementioned orthogonal test scheme was reasonable.

**3.3.2 Analysis of Variance**

**Table 10.** Analysis of variance of influencing factors of the pavement structure.

|  | **Source of variance** | Influencing factors | | | | Statistical significance |
| --- | --- | --- | --- | --- | --- | --- |
|  |  | Sum of squares | Degree of freedom f | Mean square sum S/f | F value |  |
| **Road surface deflection (mm)** | **A** | 0.020 | 2 | 0.01 | 10.00 | * |
|  | B | 0.048 | 2 | 0.024 | 24.00 | ** |
|  | C | 0.013 | 2 | 0.0065 | 6.50 |  |
|  | D(e) | 0.002 | 2 | 0.001 |  |  |
| Maximum shear stress (kPa) | A | 2.896 | 2 | 1.448 | 8.00 |  |
|  | B | 46.196 | 2 | 23.098 | 127.61 | ** |
|  | C | 117.109 | 2 | 58.5545 | 323.51 | *** |
|  | D(e) | 0.362 | 2 | 0.181 |  |  |
| Maximum tensile strain at the bottom of the layer（×10^(-6)） | A | 0.027 | 2 | 0.0135 | 3.38 |  |
|  | B | 0.104 | 2 | 0.052 | 13.00 | ** |
|  | C | 0.056 | 2 | 0.0028 | 0.70 |  |
|  | D(e) | 0.008 | 2 | 0.004 |  |  |

*** indicates highly significant, ** indicates more significant, * indicates significant.

Note: D in the source of variance is a blank column, which is the error item e.

From the F distribution table, it was determined that F_1-0.05_ (2, 2) = 19.00; F_1-0.01_ (2, 2) = 99.00; F_1-0.1_ (2, 2) = 9.00, and the road surface deflection was a characteristic of the road surface The indicators of overall stiffness and strength are shown in Table 10, F_B_=24.00>F_1-0.05_(2,2)=19.0, F_A_=10.00>F_1-0.1_(2,2)=9.00, F_C_<F_1-0.1_(2, 2)=9.00,factor B (basalt fiber reinforced the dense gradation asphalt concrete AC-20,BFAC-20) was significant, factor A (basalt fiber reinforced the gap-graded asphalt concrete SMA-16,BFSMA-16) was slightly significant, and factor C (basalt fiber reinforced asphalt-treated permeable base ATB-25,BFATB-25) had no effect on the test results; under the maximum shear stress index, In Table 10, F_C_=323.51>F_1-0.01_(2,2)=99.00, F_B_=127.61>F_1-0.01_(2,2)=99.00, and F_A_<F_1-0.1_(2,2)=9.00, Factor C (BFATB-25) was highly significant, factor B (BFAC-20) was slightly significant, and factor A (BFSMA-16) had no effect on the test results. Under the maximum tensile strain index at the bottom of the layer, Table 10 shows that F_C_=13.00>F_1-0.1_(2,2)=9.00, F_A_<F_1-0.1_(2,2)=9.00, and F_C_<F_1-0.1_(2,2)=9.00, which determine factor B (BFAC-20) significantly, while factor A (BFSMA-16) and factor C (BFATB-25) had no effect on the test results.

In summary, according to the results of the range analysis and variance analysis, there were two optimal solutions. Scheme 1 shows that the optimal dosage of the upper layer was 0.3% BFSMA-16, the optimal dosage of the middle surface layer was 0.1% BFAC-20, and the optimal dosage of the lower layer was 0.3% BFATB-25. The Scheme 2 was that the optimal dosage of the upper layer was 0.3% BFSMA-16, the optimal dosage of the middle surface layer was 0.3% BFAC-20, and the optimal dosage of the lower layer was 0.3% BFATB-25.

**3.4 Optimization analysis of basalt fiber asphalt pavement structure combination**

In order to better optimize the pavement structure combination analysis, we will analyze the performance of the four pavement structures in terms of fatigue cracking life, shear stress under different axle loads, and strong base thin surface. The following Figure 11, 12, 13 and 14 shows the four pavement structures selected for this paper.

Figure 11. Traditional pavement structure

Figure 12. Scheme 1

Figure 13. Scheme 2

Figure 14. Pavement structure with strong base and thin surface

**3.4.1 Optimization analysis of pavement structure combination based on fatigue cracking life**

Three kinds of pavement structures (traditional pavement structure, scheme 1, and scheme 2 pavement structures) were selected, and fatigue cracking calculations were conducted to determine the optimal pavement structure. The simplified model for calculating the fatigue cracking life of the asphalt mixture was:

$N_{f1}=2.78\times10^{18}\times\left(\frac{1}{\varepsilon_{a}}\right)^{3.97}\left(\frac{1}{E_{a}}\right)^{1.58}$ (3)

ε_a_---The tensile strain at the bottom of the asphalt mixture layer (×10^(-6)), the tensile strain at the bottom of the traditional pavement structure was 6.001, and the tensile strains at the bottom of scheme 1 and scheme 2 were 7.300 and 7.562, respectively.

E_a_---The dynamic compression modulus (MPa) of the asphalt mixture at 20 °C, the dynamic compression modulus of the traditional pavement structure was 9393 MPa, and that of scheme 1 and scheme 2 was 13124 MPa*.*

Through the calculation using Formula (1), the fatigue cracking life of the asphalt mixture layer of the traditional pavement structure was N_f0_=4.2442×10^11 axles, the fatigue cracking life of the asphalt mixture layer of the pavement structure of Scheme 1 was N_f1_=2.2973×10^11 axles, and the fatigue cracking life of the asphalt mixture layer of the pavement structure of Scheme 2 was N_f2_=1.9972×10^11 axles.

According to the asphalt pavement structure with different fiber contents, a simplified model could be used to calculate the fatigue crack life of the asphalt mixture. For tensile strain at the base of the same asphalt mix, the smaller the moduli of the top and middle layers were, the smaller the tensile strain and the longer the fatigue life. However, in asphalt mixtures with different fiber contents, increasing the fiber content did not improve the fatigue life of the lower layer.

From the perspective of fiber content, the fiber asphalt of the lower layer shows good fatigue performance when the content is 0.3%. This also shows that the modulus value of the base asphalt mix should be controlled within a certain range rather than blindly increasing the fiber content or taking only the best content in each layered structure to obtain the best performance of the overall structure. In addition, it is not advisable to blindly choose the method of adding the best fiber material to the top and middle layers only to improve the high- or low-temperature stability of the asphalt mix.

To improve the other properties of the asphalt pavement by adding fibers, rather than improving the fatigue life, adding fibers to the lower layer would be the wrong choice because the fatigue life of the original asphalt pavement would be already optimal. However, to increase the modulus of the upper and middle layers, it is obvious that the fatigue life of the asphalt pavement would be greatly reduced if the lower layer is not changed accordingly. For the 0.3% basalt fiber reinforced asphalt mixture, under the fatigue life control index, the reasonable fiber content range of the top and middle layers would be 0-0.3% for the top layer and 0-0.1% for the middle layer. Therefore, the pavement structure of the first scheme was selected, i.e., the optimum content of the top layer was 0.3% basalt fiber reinforced the gap-graded asphalt concrete SMA-16 (BFSMA-16), the optimum content of the middle layer was 0.1% basalt fiber reinforced the dense gradation asphalt concrete AC-20(BFAC-20), and the optimum content of the bottom layer was 0.3% basalt fiber reinforced asphalt-treated permeable base ATB-25 (BFATB-25).

***3.4.2. Optimization analysis of pavement structure based on shear stress under different axle loads***

The shear stress of the two optimal schemes was analyzed. The maximum shear stresses of the traditional pavement structure, scheme 1, and scheme 2 are shown in Table 11.

**Table 11.** Maximum shear stress of three kinds of pavements under different axle loads.

| **Axle load P/kN** | **Axial pressure/MPa** | **Equivalent circle radius/m** | **Maximum shear stress/kPa** | | |
| --- | --- | --- | --- | --- | --- |
|  |  |  | **Traditional pavement structure** | **Scheme 1** | **Scheme 2** |
| 100 | 0.707 | 0.1065 | 176.9 | 161.60 | 166.20 |
| 120 | 0.765 | 0.1117 | 193.8 | 175.30 | 179.70 |
| 150 | 0.830 | 0.1200 | 209.4 | 187.30 | 189.60 |
| 200 | 0.906 | 0.1326 | 226.3 | 199.80 | 198.80 |

**Figure 15.** Maximum shear stress under different axle loads.

Figure 15 shows that under different axle loads, the maximum shear stress occurred at the single-circle load center. When the axle load was 100 kN, the maximum shear stress of scheme 1 was reduced by 9.4% compared with the traditional pavement structure. The maximum shear stress of the traditional pavement structure was reduced by 6.4%, and the maximum shear stress of Scheme 1 was reduced by 2.8% compared with Scheme 2. When the axle load was 120 kN, the maximum shear stress of Scheme 1 was reduced by 10.5% compared with the traditional pavement structure. The maximum shear stress of scheme 2 was reduced by 7.8% compared with the traditional pavement structure, and the maximum shear stress of scheme 1 was reduced by 2.5% compared with scheme 2; when. When the axle load was 150 kN, the maximum shear stress of scheme 1 was reduced by 11.8% compared with the traditional pavement structure, the maximum shear stress of scheme 2 was reduced by 10.4% compared with the traditional pavement structure, and the maximum shear stress of scheme 1 was reduced by 1.2% compared with scheme 2. When the axle load was 200 kN, the maximum shear stress of scheme 1 compared with the traditional pavement structure was X. The stress was reduced by 13.2%, the maximum shear stress of scheme two was reduced by 13.8% compared with the traditional pavement structure, and the maximum shear stress of scheme two was reduced by 0.5% compared with scheme one. Therefore, in the case of overloading, the optimal solution obtained from the comparison was Scheme 1 (that is, the optimal dosage of the upper layer was 0.3% BFSMA-16, and the optimal dosage of the middle surface layer was 0.1% BFAC-20, and the optimal dosage of the lower layer was 0.3% BFATB-25).

***3.4.3. Optimization analysis of pavement structure combination based on the strong foundation and thin semi-rigid base***

Three kinds of pavement structures (traditional pavement structure, pavement structure with basalt fiber added, pavement structure with strong foundation and thin surface) were considered, and after calculations, the pavement surface deflection, maximum shear stress, and maximum tensile strain at the bottom of the layer are shown in Figures 16, 17, and 18.

**Figure 16.** Surface deflection of three pavement structures.

**Figure 17.** Pavement deflection of **t**hree pavement structures.

**Figure 18.** Pavement deflection of three pavement structures.

In Figure. 16 that compared with the traditional pavement structure, the surface deflection of the pavement structure with basalt fiber decreased by 2.2%, and the pavement structure with a strong base and thin surface increased by 0.1%. In Figure. 17, compared with the traditional pavement structure, the maximum shear stress of the pavement structure with basalt fiber decreased by 6.4%, and the pavement structure with a strong base and thin surface increased by 22.4%. According to Figure 18, compared to the traditional pavement structure, the maximum tensile strain at the bottom of the pavement structure with basalt fiber increased by 21.6%, and the maximum tensile strain at the bottom of the pavement structure with a strong base and thin surface increased by 9.2%. The higher the tensile strain at the bottom of the layer was, the greater the strength and the ability of the material to withstand large deformation without breaking. Therefore, through comprehensive consideration and analysis, we could also use the pavement structure that consists of a strong base and thin surface.

**4. Conclusions**

The use of basalt fiber in asphalt mixtures is a common practice, yet limited research addresses its impact on the mechanical properties of asphalt pavement materials, particularly in relation to functional requirements across different pavement layers. This project examines the influence of basalt fiber content on asphalt pavement mechanical properties using three-dimensional finite element software ABQUAS. The investigation covers upper-layer resistance to low-temperature cracking, middle-layer resistance to rutting, and lower-layer resistance to fatigue cracking. The study's findings are summarized as follows:

(1) With the change in basalt fiber content, the deflection of the road surface and the maximum tensile strain at the bottom of the asphalt layer of the nine asphalt pavement structures were located at the single-circle load center, while the position of the maximum shear stress was located at the single-circle load center or the single-circle load at the outer edge.

(2) Combined with the orthogonal test analysis based on fatigue life and overload, the optimum pavement structure was determined by calculation and analysis as 0.3% BFS-MA-16 for the upper layer, 0.1% BFAC-20 for the middle layer and 0.3% BFATB-25 for the lower layer. This solution can be used for the pavement surface materials of high-grade highways or the level crossing pavement surface of urban city roads.

(3) Combined with the orthogonal test, it was determined that the pavement structure of the strong base and thin-surface semi-rigid base was 4 cm BFSMA-16 (fiber content was 0.3%), 8 cm BFSMA-20 (fiber content was 0.1%), 44 cm cement-stabilized gravel base, and 18 cm cement-stabilized gravel subbase. The pavement surface material program is primarily designed for structural surfacing, specifically targeting long-life pavements currently under investigation in China.

**Author Contributions**

**Conceptualization:** Xiangbing Xie, Yahui He and Chenchen Liu;

**Data curation:** Yahui He ;

**Formal analysis:** Xiangbing Xie, Kaiwei Wang and Yahui He;

**Funding acquisition:** Xiangbing Xie , Jinggan Shao ;

**Investigation:** Xiangbing Xie, Kaiwei Wang and Yahui He;

**Methodology:** Kaiwei Wang, Xiangbing Xie and Huixia Li;

**Project administration:** Xiangbing Xie;

**Resources:** Xiangbing, Yahui He;

**Software:** Kaiwei Wang and Yahui He and Chenchen Liu;

**Supervision:** Xiangbing Xie ;

**Validation:** Xiangbing Xie and Zhezhe Fan;

**Visualization:** Xiangbing Xie, Kaiwei Wang;

**Writing—original draft preparation:** Xiangbing Xie, Yahui He;

**Writing—review and editing:** Zhezhe Fan, Huixia Li and Yahui He;

All authors have read and agreed to the published version of the manuscript.

**Funding**

The authors appreciate the financial support from the Youth Research Funds Plan of Zhengzhou University of Aeronautics (Grant No. 23HQN01007), Research on Application Technology and Equipment of Sprayed Basalt Fiber Reinforced Concrete (Grant No. 2020J-2-12), Research on Key Technologies of Application of Basalt Fiber and its Products in Highway Engineering (Grant No. 2021J5), the National Natural Science Foundation of China (Grant No. 51378474), Fund of Leading Talent in Science and Technology Innovation (Grant No. 194200510015), Science and Technology Department of Henan Province (Grant No. 192102210047), and Scientific Research and Development Project of Zhengzhou Lutong Highway Construction Co., Ltd. (Grant No. 2021JK-11).

**Data Availability Statement**

Data is available on request from the corresponding author.

**Conflicts of Interest**

The authors declare no conflicts of interest.

References

1. Álvarez, A.E.; Walubita, L.F.; Sánchez, F. Using fracture energy to characterize the hot mix asphalt cracking resistance based on the direct-tensile test. Rev. Fac. Ing. 2012, 20, 126–137.
2. Xu, W.Z. Research on Cracking Behavior of High Dosage Hot Reclaimed Asphalt Mixture Based on Digital Speckle Method. Master’s Thesis, Shenyang University of Civil Engineering and Architecture, Shenyang, China, 2021.
3. Alfalah, A.; Offenbacker, D.; Ali, A.; Decarlo, C.; Lein, W.; Mehta, Y.; Elshaer, M. Assessment of the impact of fiber types on the performance of fiber-reinforced hot mix asphalt. Transp. Res. Rec. 2020, 2674, 337–347.
4. Sheng, Y.P.; Zhang, B.; Yan, Y.; Li, H.B.; Chen, Z.J.; Chen, H.X. Laboratory Investigation on the Use of Bamboo Fiber in Asphalt Mixtures for Enhanced Performance. Arab. J. Sci. Eng. 2019, 44, 4629–4638.
5. Rathore, M.; Haritonovs, V.; Zaumanis, M. Performance evaluation of warm asphalt mixtures containing chemical additive and effect of incorporating high reclaimed asphalt content. Materials 2021, 14, 3793.
6. Zheng, D.; Qian, Z.D.; Li, P.; Wang, L.B. Performance evaluation of high-elasticity asphalt mixture containing inorganic nanotitanium dioxide for applications in high altitude regions. Constr. Build. Mater. 2019, 199, 594–600.
7. Guo, Q.; Wang, H.; Gao, Y.; Jiao, Y.; Dong, Z. Investigation of the low-temperature properties and cracking resistance of fiber-reinforced asphalt concrete using the DIC technique. Eng. Fract. Mech. 2020, 229, 106951.
8. Zhang, K.; Liu, Y.; Nassiri, S.; Li, H.; Englund, K. Performance evaluation of porous asphalt mixture enhanced with high dosages of cured carbon fiber composite materials. Constr. Build. Mater. 2021, 274, 122066.
9. Luo, D.; Khater, A.; Yue, Y.; Abdelsalam, M.; Zhang, Z. The performance of asphalt mixtures modified with lignin fiber and glass fiber: A review. Constr. Build. Mater. 2019, 209, 377–387.
10. Alfalah, A.; Offenbacker, D.; Ali, A.; Mehta, Y. Evaluating the impact of fiber type and dosage rate on laboratory performance of Fiber-Reinforced asphalt mixtures. Constr. Build. Mater. 2021, 310, 125217.
11. Jamshaid, H.; Mishra, R. A green material from rock: Basalt fiber—A review. J. Text. I. 2015, 107, 923–937.
12. Lou, K.; Kang, A.; Xiao, P.; Wu, Z.; Li, B.; Wang, X. Effects of basalt fiber coated with different sizing agents on performance and microstructures of asphalt mixture. Constr. Build. Mater. 2021, 266, 121155.
13. Yan, L.; Chu, F.; Tuo, W.; Zhao, X.; Wang, Y. Review of research on basalt fibers and basalt fiber-reinforced composites in China (I): Physicochemical and mechanical properties. Polym. Polym. Compos. 2020, 29, 1612–1624.
14. Sha, A.; Liu, Z.; Jiang, W.; Qi, L.; Hu, L. Advances and development trends in eco-friendly pavements. J. Road Eng. 2021, 1, 1–42.
15. Wang, L. S, Key technology of basalt fiber SMA modified asphalt pavement construction. Hebei Province, Hebei Road and Bridge Group Co.2014-08-18.
16. Thanh, D. V.; Feng, C. P., Study on Marshall and Rutting test of SMA at abnormally high temperature. Construction and Building Materials 2013, 47.
17. Zhang Y. J., Zhai X. J , Ma Y. Q., Optimization Design and Performance Evaluation of Basalt Fiber Asphalt Mixture [J]. Journal of Highway and Transportation Research and Development: Application Technology Edition, 2014(3).
18. Zhao Y. S., Li H T., Influence of Basalt Short Fiber on Asphalt Concrete Performance [J]. Journal of Highway and Transportation Research and Development, 2012, 29(9):38-42.
19. Wu B. W., Experimental Study on the Performance of Basalt Fiber-Reinforced Asphalt Mixture [D]. Yangzhou University, 2013.
20. Cao, Q. Y.; Gao, K. K., Study on the effect of different fiber dosing on the performance of asphalt mixes. Modern Transportation Technology 2022, 19, (04), 17-20.
21. Li, Z.; Shen, A.; Wang, H.; Guo, Y.; Wu, H., Effect of basalt fiber on the low-temperature performance of an asphalt mixture in a heavily frozen area. Construction and Building Materials 2020, 253, (C)
22. Zhao, H.; Guan, B.; Xiong, R.; Zhang, A., Investigation of the Performance of Basalt Fiber Reinforced Asphalt Mixture. Applied Sciences 2020, 10, (5).
23. Fan, W. X.; Kang, H. G.; Zheng, Y. X., Experimental study on the road performance of basalt fiber modified asphalt mixtures (in English). Journal of Southeast University(English Edition) 2010, 26, (04), 614-617.
24. Liu, C, Effect of basalt fibers on the road performance of asphalt mixtures. Jiangxi Building Materials 2022, (10), 27-29+32.
25. Ma, F.; Yang, Y.; Ji, X.; Guo, X. L.; Yi, S. F., Mechanical and fatigue properties of basalt fiber asphalt mixes. Journal of Xi'an University of Technology 2022, 42, (04), 393-400.
26. Zeng, Z. Y. Performance and structural analysis of basalt fiber asphalt pavement. Master, Zhejiang University, 2013
27. Guo, F. C.; Li, R.; Lu, R. H.; Bi, Y. Q; He, H. Q, Evaluation of the Effect of Fiber Type, Length, and Content on Asphalt Properties and Asphalt Mixture Performance. Materials 2020, 13, (7)
28. Pirmohammad, S.; Amani, B.; Shokorlou, Y. M., The effect of basalt fibres on fracture toughness of asphalt mixture. Fatigue & Fracture of Engineering Materials & Structures 2020, 43, (7)
29. Li H. B.,Wei R. X. Functional analysis of semi-rigid base asphalt pavement surface layers[J]. Traffic World(Transportation. Vehicle),2012,(09):172-174.
30. Tan Y. Q., Yao L., Wang H. P., et al. Analysis of early damage layers and countermeasures of asphalt pavement structure[J]. Journal of Highway and Transportation Research and Development, 2012, 29(5):13-29.
31. Du X. Functional zoning and structural optimization design of semi-rigid base layer of asphalt pavement [D]. Chang'an University,2012.
32. Ma Q. W.. Structural research on durable asphalt pavement based on layer function consideration [D]. Chang'an University, 2010.
33. Zhang H. Z., Zang G. S., Sun L. J.. Analysis of dynamic strain response of asphalt pavement with semi-rigid base layer[J]. Transportation Science and Engineering,2017,33(01):12-17.
34. Fan Q. Research on combined base layer asphalt pavement structure and its mix design based on layer function[D]. Chongqing Jiaotong University.
35. Fan, Z. Z., Research on Prediction Model of Ruts on Asphalt Pavement at Grade Crossings Based on Dynamic Modulus" . Master's Thesis, Xi'an University of Architecture and Technology, 2016.
36. Wang, X. Design and optimization of basalt fiber asphalt mixes based on structural layer function. M.S., Harbin Institute of Technology, 2018.
37. Liu, Z. L.; Chen X. M.; Jiao, H. Z.; Wang, Y. F.; Chen, F. B.; Yang, Y. X, Optimization of basalt fiber reinforced concrete mix ratio based on orthogonal design. Concrete and Cement Products 2017, (10), 51-55.
38. Lin, J. C.; Wu, Y.; Wang, X. Analysis of structural stress characteristics of combined asphalt pavement under moving load. Si-no-Foreign Highway 2022, 42, (01), 43-47.
39. Luo, K.; Chen, J, Study on the factors influencing the performance index of basalt fiber concrete based on orthogonal test. Hunan Transportation Science and Technology 2016, 42, (04), 41-43+85,
40. Yan, J.; Jingchen, Y.; Guilai, W.; Jianlong, Z., Study on pavement performance of basalt fiber asphalt concrete under different pavement environment. Journal of Physics: Conference Series 2020, 1654, (1).
41. Gao, C. M.; Han, S.; Chen, S.; Li, H., Research on Basalt Fiber Asphalt Concrete's Low Temperature Performance. Applied Mechanics and Materials 2014, 2973, (505-506).
42. Gao, C. M.; Han, S.; Zhu, K. X.; Wang, Z. Y., Research on Basalt Fiber Asphalt Concrete's High Temperature Performance. Applied Mechanics and Materials 2014, 2973, (505-506).
43. Chen, Y. Z.; Li, Z. X., Study of Road Property of Basalt Fiber Asphalt Concrete. Applied Mechanics and Materials 2012, 2080, (238-238)
44. Jiu ,X. Y.; Wang Y.;Wu,Z.G.;Xiao,P.,High-Temperature Performance Evaluation of Asphalt Mixtures by Adding Short-Chopped Basalt Fiber.Buildings,2023,13,13020370.
45. Occupation Standard of the People’s Republic of China JTG D50; Specifications for Design of Highway Asphalt Pavements,
46. Ministry of Transport of the People’s Republic of China: Beijing, China, 2017.
47. Zhang,M.J.;Shi,Y.;Du,J.,The Influences of Surface Shear Stress Peak Value of Sem-i Rigid Base Course Asphalt Pavement Structure,Journal of Shenyang Architecture University,2007,23(2):187-190.
48. Hui,Y.X.; Men,G.Y.;Xiao,P.;Tang,Q,Recent Advances in Basalt Fiber Reinforced Asphalt Mixture for Pavement Applications,Materials,2022, 15, 15196826..
