# Supplementary material for: Optimization of the dosage of chopped basalt fibers in asphalt pavement surface course materials for semi-rigid base with functional requirements
Source: PLoS One. 2024 Jul 29;19(7):e0307438. doi: 10.1371/journal.pone.0307438 (PMC11285914; doi:10.1371/journal.pone.0307438)
Supplement: Attachment — Submitted filename: Response to Reviewer 2.doc [file pone.0307438.s002.doc]

**Response to Reviewer 2 Comments**

1.Tests for coarse aggregate used in preparing the SMA mixture are not included in the research, so please add it as it is an essential factor in preparing the mixture.

Response 1: Thank you very much for the editor's suggestions on my paper. For the results of the coarse aggregate tests used to prepare the SMA mix, I have added to Section 2.1.3.

***2.1.3 Aggregate***

The high-quality limestone from the material yard of Hongtu Highway in Inner Mongolia is used, and the mineral powder is calcium carbonate powder. According to the requirements of ' Highway Engineering Aggregate Test Procedure ' ( JTG E42-2005 ), the performance indexes of different coarse and fine aggregates were tested. The test results are shown in Table 3 ~ 5.

| **Table 3.** Technical index of coarse aggregate | | | | | | |
| --- | --- | --- | --- | --- | --- | --- |
| Inspection project | Unit | Coarse aggregate test results | | | Specification  requirement | Test method |
|  |  | 20~40mm | 10~20mm | 5~10mm |  |  |
| Aggregate crushing value | % | 15.5 | —— | —— | ≤28 | T0316-2005 |
| Aggregate abrasion loss | % |  | 10.6 |  | ≤30 | T0317-2005 |
| Apparent relative density of aggregates | —— | 2.752 | 2.731 | 2.787 | ≤2.5 |  |
| Relative density of aggregate gross volume | —— | 2.736 | 2.702 | 2.737 | Measured value | T0304-2005 |
| Aggregate water absorption | % | 1.12 | 1.65 | 1.61 | ≤3.0 |  |
| Firmness | % | 6.8 | —— | —— | ≤12 | T0314-2005 |
| Flat elongated particles content | % | 16 5.5 | 15 | 15 | Measured value | T0312-2005 |
|  | % | 5.5 | 5.1 | 5.0 | Required value |  |
| Particle content less than 0.075 mm ( water washing method ) | % | 0.12 | 0.31 | 0.43 | ≤1 | T0310-2005 |

2.The mechanism for mixing and preparing the mixture according to the specification is explained accurately. If the researcher uses models from previous research belonging to him in the analysis, please mention them.

Response 2: Thank you very much for the editor's suggestions on my paper. We have described the preparation mechanism of the mixture, which is labeled in blue in the text.

***2.1.4 mix design***

According to the specification and the test results of raw materials, the gradation design curve is fitted, and the aggregate gradation of SMA-16, AC-20 and ATB-25 is determined. The gradation is as shown in Table 6,7,8.

|  | | **Table 6.** Aggregate gradation of SMA-16 | | | | | | | | | | | | | | | | | | | | | | | | | | | | | | | |
| --- | --- | --- | --- | --- | --- | --- | --- | --- | --- | --- | --- | --- | --- | --- | --- | --- | --- | --- | --- | --- | --- | --- | --- | --- | --- | --- | --- | --- | --- | --- | --- | --- | --- |
|  | | Particle  size/mm | | | | The mass percentage passing through the following sieves（%） | | | | | | | | | | | | | | | | | | | | | | | | | | | |
|  | |  |  |  |  | 19 | | | 16 | | | 13.2 | | | 9.5 | | | 4.75 | | | 2.36 | | | 1.18 | | | 0.6 | | | 0.3 | | 0.15 | 0.075 |
|  | | Upper gradation limit/% | | | | 100 | | | 100 | | | 85 | | | 65 | | | 32 | | | 24 | | | 22 | | | 18 | | | 15 | | 14 | 12 |
|  | | Lower gradation limit/% | | | | 100 | | | 90 | | | 65 | | | 45 | | | 20 | | | 15 | | | 14 | | | 12 | | | 10 | | 9 | 8 |
|  | | Gradation median /% | | | | 100 | | | 95 | | | 75 | | | 55 | | | 26 | | | 19.5 | | | 18 | | | 15 | | | 12.5 | | 11.5 | 10 |
|  | | Synthetic gradation/% | | | | 100 | | | 95.6 | | | 74.2 | | | 53.6 | | | 24.5 | | | 18.5 | | | 17 | | | 13.2 | | | 11.5 | | 10 | 10 |
|  | **Table 7.** Aggregate gradation of AC-20 | | | | | | | | | | | | | | | | | | | | | | | | | | | | | | | | |
|  | Particle  size/mm | | | | The mass percentage passing through the following sieves（%） | | | | | | | | | | | | | | | | | | | | | | | | | | | | |
|  |  |  |  |  | 26.5 | | | 19 | | | 16 | | | 13.2 | | | 9.5 | | | 4.75 | | | 2.36 | | | 1.18 | | | 0.6 | | 0.3 | 0.15 | 0.075 |
|  | Upper gradation limit/% | | | | 100 | | | 100 | | | 92 | | | 80 | | | 72 | | | 56 | | | 44 | | | 33 | | | 24 | | 17 | 13 | 7 |
|  | Lower gradation limit/% | | | | 100 | | | 90 | | | 78 | | | 62 | | | 50 | | | 26 | | | 16 | | | 12 | | | 8 | | 5 | 4 | 3 |
|  | Gradation median/% | | | | 100 | | | 95 | | | 85 | | | 71 | | | 61 | | | 41 | | | 30 | | | 22.5 | | | 16 | | 11 | 8.5 | 5 |
|  | Synthetic gradation/% | | | | 100 | | | 94 | | | 84 | | | 73 | | | 58 | | | 41 | | | 29 | | | 22 | | | 15 | | 12 | 8 | 4 |
| **Table 8.** Aggregate gradation of ATB-25 | | | | | | | | | | | | | | | | | | | | | | | | | | | | | | | | | |
| Particle  size/mm | | | The mass percentage passing through the following sieves（%） | | | | | | | | | | | | | | | | | | | | | | | | | | | | | | |
|  |  |  | 31.5 | 26.5 | | | 19 | | | 16 | | | 13.2 | | | 9.5 | | | 4.75 | | | 2.36 | | | 1.18 | | | 0.6 | | | 0.3 | 0.15 | 0.075 |
| Upper gradation limit/% | | | 100 | 100 | | | 80 | | | 68 | | | 62 | | | 52 | | | 40 | | | 32 | | | 25 | | | 18 | | | 14 | 10 | 6 |
| Lower gradation limit/% | | | 100 | 90 | | | 60 | | | 48 | | | 42 | | | 32 | | | 20 | | | 15 | | | 10 | | | 8 | | | 5 | 3 | 2 |
| Gradation median/% | | | 100 | 95 | | | 70 | | | 58 | | | 52 | | | 42 | | | 30 | | | 23.5 | | | 17.5 | | | 13 | | | 9.5 | 6.5 | 4 |
| Synthetic gradation/% | | | 100 | 96.5 | | | 69.2 | | | 57.5 | | | 50.3 | | | 43.5 | | | 32 | | | 22.8 | | | 15 | | | 11.5 | | | 9 | 6.5 | 5.2 |

***2.1.5 Determination of optimum oil-stone ratio***

The mixture ratio design of SMA-16 and AC-20 asphalt mixture adopts Marshall test method. The two sides of the specimen are compacted 75 times, and the oil-stone ratio is 4 %, 4.5 %, 5.0 %, 5.5 % and 5.0 % respectively. The mix design of asphalt mixture of ATB-25 adopts Marshall test method. The double sides of the specimen are compacted 75 times, and the oil-stone ratio is 3.0 %, 3.3 %, 3.6 %, 3.9 % and 4.2 % respectively. The volume performance and Marshall test index of the specimen under the design compaction times were measured, so as to determine the best oil-stone ratio according to the design standard. The volume characteristics of asphalt mixture under each asphalt-aggregate ratio are shown in Table 9,10 and 11.

| **Table 9.** Volumetric properties of SMA-16 mixes with different oil/gravel ratios | | | | | | | | | | | | | | | |
| --- | --- | --- | --- | --- | --- | --- | --- | --- | --- | --- | --- | --- | --- | --- | --- |
| Oil-rock ratio (%) | | | Marshall double-sided each hit 75 times | | | | | | | | | | | | |
|  |  |  | Gross bulk density  (g/cm^3^) | Void ratio(%) | Mineral Gap Ratio(%) | | Asphalt saturation  (%) | | | | | Stability  (kN) | | Flow value  (mm) | |
| 4.8 | | | 2.460 | 4.0 | 14.6 | | 72.3 | | | | | 9.53 | | 38.3 | |
| 5.1 | | | 2.462 | 3.9 | 15.5 | | 74.5 | | | | | 10.17 | | 36.3 | |
| 5.4 | | | 2.469 | 3.3 | 15.5 | | 79 | | | | | 8.26 | | 36.7 | |
| 5.7 | | | 2.461 | 3.1 | 16 | | 80.3 | | | | | 8.73 | | 30.23 | |
| 6.0 | | | 2.465 | 3.0 | 16.2 | | 82. | | | | | 8.85 | | 29.36 | |
| **Table 10.** Volumetric properties of AC-20 mixes with different oil/gravel ratios | | | | | | | | | | | | | | | |
| Oil-rock ratio (%) | Marshall double-sided each hit 75 times | | | | | | | | | | | | | | |
|  | Gross bulk density  (g/cm^3^) | | | Void ratio(%) | | Mineral Gap Ratio(%) | | | Asphalt saturation  (%) | | Stability  (kN) | | Flow value  (mm) | | |
| 4.2 | 2.487 | | | 6.5 | | 16.2 | | | 59.6 | | 12.05 | | 26.0 | | |
| 4.5 | 2.506 | | | 5.4 | | 15.8 | | | 65.8 | | 13.60 | | 27.8 | | |
| 4.8 | 2.513 | | | 4.7 | | 15.8 | | | 70.0 | | 13.21 | | 21.2 | | |
| 5.1 | 2.516 | | | 4.5 | | 14.8 | | | 71.2 | | 13.56 | | 24.6 | | |
| 5.4 | 2.523 | | | 4.2 | | 14.5 | | | 73.5 | | 13.85 | | 23.5 | | |
| **Table 11.** Volumetric properties of ATB-25 mixes with different oil/gravel ratios | | | | | | | | | | | | | | | |
| Oil-rock ratio (%) | | Marshall double-sided each hit 75 times | | | | | | | | | | | | | |
|  |  | gross bulk density  (g/cm^3^) | | void ratio(%) | | Mineral Gap Ratio(%) | | Asphalt saturation  (%) | | Stability  (kN) | | | | | Flow value  (mm) |
| 3.1 | | 2.356 | | 6.9 | | 15.3 | | 54.7 | | 6.65 | | | | | 1.54 |
| 3.4 | | 2.371 | | 5.9 | | 15.0 | | 60.5 | | 7.74 | | | | | 2.74 |
| 3.7 | | 2.387 | | 4.9 | | 14.7 | | 66.8 | | 8.48 | | | | | 3.00 |
| 4.0 | | 2.383 | | 4.6 | | 15.0 | | 69.5 | | 8.79 | | | | | 3.04 |
| 4.3 | | 2.386 | | 4.1 | | 15.2 | | 73.2 | | 8.35 | | | | | 3.13 |

According to the Marshall test results, as shown in Table 9,10,11, the optimum asphalt-aggregate ratio of SMA-16 asphalt mixture is 5.4 %, the optimum asphalt-aggregate ratio of AC-20 asphalt mixture is 4.8 %, and the optimum asphalt-aggregate ratio of ATB-25 asphalt mixture is 3.7 %.

3.Preparing samples according to the specification of the mixture not included in the research paper. Please add them carefully, especially for SMA.

Response 3: Thank you very much for the editor's suggestions on my paper. I have added the technical specifications for the prepared samples of the mixtures, which are indicated in blue font in the text.

***2.1.1 Asphalt***

In this study, the SBS I-C modified road petroleum asphalt of Fuwei Chemical Co., Ltd.was used, and its performance indicators were as follows:

**Table 1.** Performance indicators of modified asphalt.

| **Parameters** | **Requirements** | **Experimental results** | **Experimental method** |
| --- | --- | --- | --- |
| Penetration (25%)/0.1 mm | 60-80 | 71 | GB/T 4509 |
| Softening point/ °C | ≥ 60 | 79 | GB/T 4507 |
| Ductility (5℃,5 cm/min)/cm | ≥ 30 | 34 | GB/T 4508 |
| Penetration index PI | -1.5-1 | 0.16 |  |
| Solubility/% | ≥ 99.0 | 99.5 | GB/T 11148 |
| Flashpoint/ °C | ≥ 230 | 281 | GB/T267 |

***2.1.2 Fiber***

Chopped 6mm/17 μm basalt fiber was used in this experiment, and its various performance indicators are shown in Table 2.

**Table 2. Performance indexes of chopped basalt fiber.**

| **Parameters** | **Requirements** | **Experimental results** |
| --- | --- | --- |
| Breaking strength/MPa | ≥ 1200 | 1260 |
| Elongation at break/% | ≤ 3.1 | 2.65 |
| Oil absorption/% | ≥ 50 | 53 |
| Flammability content% | 0.1-1.0 | 0.5 |
| Moisture content% | ≤ 0.2 | 0.14 |
| Flammability | open flame otherwise | open flame otherwise |

***2.1.3 Aggregate***

The high-quality limestone from the material yard of Hongtu Highway in Inner Mongolia is used, and the mineral powder is calcium carbonate powder. According to the requirements of ' Highway Engineering Aggregate Test Procedure ' ( JTG E42-2005 ), the performance indexes of different coarse and fine aggregates were tested. The test results are shown in Table 3 ~ 5.

| **Table 3.** Technical index of coarse aggregate | | | | | | | | | | | | | | | | | |
| --- | --- | --- | --- | --- | --- | --- | --- | --- | --- | --- | --- | --- | --- | --- | --- | --- | --- |
| Inspection project | | | Unit | Coarse aggregate test results | | | | | | | | | | Specification  requirement | | | Test method |
|  |  |  |  | 20~40mm | | | 10~20mm | | | 5~10mm | | | |  |  |  |  |
| Aggregate crushing value | | | % | 15.5 | | | —— | | | —— | | | | ≤28 | | | T0316-2005 |
| Aggregate abrasion loss | | | % |  | | | 10.6 | | |  | | | | ≤30 | | | T0317-2005 |
| Apparent relative density of aggregates | | | —— | 2.752 | | | 2.731 | | | 2.787 | | | | ≤2.5 | | |  |
| Relative density of aggregate gross volume | | | —— | 2.736 | | | 2.702 | | | 2.737 | | | | Measured value | | | T0304-2005 |
| Aggregate water absorption | | | % | 1.12 | | | 1.65 | | | 1.61 | | | | ≤3.0 | | |  |
| Firmness | | | % | 6.8 | | | —— | | | —— | | | | ≤12 | | | T0314-2005 |
| Flat elongated particles content | | | % | 16 5.5 | | | 15 | | | 15 | | | | Measured value | | | T0312-2005 |
|  |  |  | % | 5.5 | | | 5.1 | | | 5.0 | | | | Required value | | |  |
| Particle content less than 0.075 mm ( water washing method ) | | | % | 0.12 | | | 0.31 | | | 0.43 | | | | ≤1 | | | T0310-2005 |
|  | | **Table 4.** Test results of fine aggregate | | | | | | | | | | | | | | | |
|  | | Inspection project | | | Unit | | | Test results of fine aggregate | | | | | | | | | |
|  | |  |  |  |  |  |  | 3~5 | | | Stone chips | | Specification  requirement | | | Test method | |
|  | | Apparent specific gravity | | | —— | | | 2.726 | | | 2.712 | | ≤22.5 | | | T0304-2005 | |
|  | | Mud content ( < 0.075 mm content ) | | | % | | | 1.1 | | | 2.4 | | ≤3 | | | T0333-2005 | |
| **Table 5.** Test results of mineral powder | | | | | | | | | | | | | | | | | |
| Inspection project | | | | | | Unit | | | Test results | | | Specification  requirement | | | Test method | | |
| Apparent specific gravity | | | | | | t/m^3^ | | | 2.713 | | | ≤2.5 | | | T0352-2005 | | |
| Particle size range | <0.6 | | | | | % | | | 99.84 | | | 100 | | | T0351-2005 | | |
|  | <0.15 | | | | | % | | | 91.91 | | | 90~100 | | |  |  |  |
|  | <0.075 | | | | | % | | | 71.93 | | | 75~100 | | |  |  |  |
| Hydrophilic coefficient of mineral powder | | | | | | —— | | | 0.8 | | | <1.0 | | | T0353-2005 | | |
| Plasticity index | | | | | | —— | | | 3.7 | | | <4 | | | T0354-2005 | | |

4.This type of mixture needs additives. Please indicate the type and percentages used

Response 4: Thank you very much for the editor's suggestions on my paper. I have added the grading curves of the asphalt mixes and the percentages of the various particle sizes, which are labeled in blue in the text.

***2.1.4 mix design***

According to the specification and the test results of raw materials, the gradation design curve is fitted, and the aggregate gradation of AC-16, AC-20 and ATB-30 is determined. The gradation is as shown in Table 6,7,8, and the gradation curve is shown in Figure 1,2 and 3.

|  | | **Table 6.** Aggregate gradation of SMA-16 | | | | | | | | | | | | | | | | | | | | | | | | | | | | | | | |
| --- | --- | --- | --- | --- | --- | --- | --- | --- | --- | --- | --- | --- | --- | --- | --- | --- | --- | --- | --- | --- | --- | --- | --- | --- | --- | --- | --- | --- | --- | --- | --- | --- | --- |
|  | | Particle  size/mm | | | | The mass percentage passing through the following sieves（%） | | | | | | | | | | | | | | | | | | | | | | | | | | | |
|  | |  |  |  |  | 19 | | | 16 | | | 13.2 | | | 9.5 | | | 4.75 | | | 2.36 | | | 1.18 | | | 0.6 | | | 0.3 | | 0.15 | 0.075 |
|  | | Upper gradation limit/% | | | | 100 | | | 100 | | | 85 | | | 65 | | | 32 | | | 24 | | | 22 | | | 18 | | | 15 | | 14 | 12 |
|  | | Lower gradation limit/% | | | | 100 | | | 90 | | | 65 | | | 45 | | | 20 | | | 15 | | | 14 | | | 12 | | | 10 | | 9 | 8 |
|  | | Gradation median /% | | | | 100 | | | 95 | | | 75 | | | 55 | | | 26 | | | 19.5 | | | 18 | | | 15 | | | 12.5 | | 11.5 | 10 |
|  | | Synthetic gradation/% | | | | 100 | | | 95.6 | | | 74.2 | | | 53.6 | | | 24.5 | | | 18.5 | | | 17 | | | 13.2 | | | 11.5 | | 10 | 10 |
|  | **Table 7.** Aggregate gradation of AC-20 | | | | | | | | | | | | | | | | | | | | | | | | | | | | | | | | |
|  | Particle  size/mm | | | | The mass percentage passing through the following sieves（%） | | | | | | | | | | | | | | | | | | | | | | | | | | | | |
|  |  |  |  |  | 26.5 | | | 19 | | | 16 | | | 13.2 | | | 9.5 | | | 4.75 | | | 2.36 | | | 1.18 | | | 0.6 | | 0.3 | 0.15 | 0.075 |
|  | Upper gradation limit/% | | | | 100 | | | 100 | | | 92 | | | 80 | | | 72 | | | 56 | | | 44 | | | 33 | | | 24 | | 17 | 13 | 7 |
|  | Lower gradation limit/% | | | | 100 | | | 90 | | | 78 | | | 62 | | | 50 | | | 26 | | | 16 | | | 12 | | | 8 | | 5 | 4 | 3 |
|  | Gradation median/% | | | | 100 | | | 95 | | | 85 | | | 71 | | | 61 | | | 41 | | | 30 | | | 22.5 | | | 16 | | 11 | 8.5 | 5 |
|  | Synthetic gradation/% | | | | 100 | | | 94 | | | 84 | | | 73 | | | 58 | | | 41 | | | 29 | | | 22 | | | 15 | | 12 | 8 | 4 |
| **Table 8.** Aggregate gradation of ATB-25 | | | | | | | | | | | | | | | | | | | | | | | | | | | | | | | | | |
| Particle  size/mm | | | The mass percentage passing through the following sieves（%） | | | | | | | | | | | | | | | | | | | | | | | | | | | | | | |
|  |  |  | 31.5 | 26.5 | | | 19 | | | 16 | | | 13.2 | | | 9.5 | | | 4.75 | | | 2.36 | | | 1.18 | | | 0.6 | | | 0.3 | 0.15 | 0.075 |
| Upper gradation limit/% | | | 100 | 100 | | | 80 | | | 68 | | | 62 | | | 52 | | | 40 | | | 32 | | | 25 | | | 18 | | | 14 | 10 | 6 |
| Lower gradation limit/% | | | 100 | 90 | | | 60 | | | 48 | | | 42 | | | 32 | | | 20 | | | 15 | | | 10 | | | 8 | | | 5 | 3 | 2 |
| Gradation median/% | | | 100 | 95 | | | 70 | | | 58 | | | 52 | | | 42 | | | 30 | | | 23.5 | | | 17.5 | | | 13 | | | 9.5 | 6.5 | 4 |
| Synthetic gradation/% | | | 100 | 96.5 | | | 69.2 | | | 57.5 | | | 50.3 | | | 43.5 | | | 32 | | | 22.8 | | | 15 | | | 11.5 | | | 9 | 6.5 | 5.2 |

5.The analysis is inaccurate, especially the installed drawings, as they do not correspond to the reality of the situation.

Response 5: Thank you very much for the editor's suggestions on my paper. I have made changes to this installation diagram.
